# Mediterranean Lifestyle to Promote Physical, Mental, and Environmental Health: The Case of Chile

**DOI:** 10.3390/ijerph17228482

**Published:** 2020-11-16

**Authors:** Guadalupe Echeverría, Ornella Tiboni, Loni Berkowitz, Victoria Pinto, Bárbara Samith, Andrea von Schultzendorff, Nuria Pedrals, Marcela Bitran, Chiara Ruini, Carol D. Ryff, Daniele Del Rio, Attilio Rigotti

**Affiliations:** 1 Departamento de Nutrición, Diabetes y Metabolismo, Escuela de Medicina, Pontificia Universidad Católica de Chile, Santiago CP 83300024, Chile; gecheverria@bio.puc.cl (G.E.); loniberko@gmail.com (L.B.); bpsamith@uc.cl (B.S.); npedrals@uc.cl (N.P.); 2 Centro de Nutrición Molecular y Enfermedades Crónicas, Escuela de Medicina, Pontificia Universidad Católica de Chile, Santiago CP 83300024, Chile; otiboni@uc.cl (O.T.); vspinto@uc.cl (V.P.); andreavonsch@gmail.com (A.v.S.); mbitran@med.puc.cl (M.B.); 3 Centro de Educación Médica y de Ciencias de la Salud, Escuela de Medicina, Pontificia Universidad Católica de Chile, Santiago CP 83300024, Chile; 4 Department of Psychology, University of Bologna, 40126 Bologna, Italy; chiara.ruini@unibo.it; 5 Institute on Aging and Department of Psychology, University of Wisconsin-Madison, Madison, WI 53706-1611, USA; cryff@wisc.edu; 6 Department of Food and Drugs, University of Parma, 43121 Parma, Italy; daniele.delrio@unipr.it

**Keywords:** diet, Mediterranean, physical health, psychological well-being, sustainability, chronic diseases, Chile

## Abstract

Chile is currently experiencing a progressive epidemiological transition towards chronic diseases. In this country, >50% of annual deaths are attributed to cardiovascular disease and cancer. Moreover, health surveys have shown high prevalence of obesity, diabetes, hypertension, and elevated cardiovascular disease risk. In addition, mental health issues are also frequent among Chilean adults. On the other hand, the agri-food system contributes to 21–37% of greenhouse gases emissions worldwide. Overall, current health and food chain situation calls out for design and implementation of evidence-based feasible and effective nutritional interventions needed to promote physical and mental health along with addressing food sustainability in Chile. Nowadays, the Mediterranean diet is recognized as one of the healthiest dietary patterns based on observational and interventional studies linked to a wide variety of health outcomes. However, a Mediterranean lifestyle goes well beyond food intake: it includes promotion of psychosocial resources, community life as well as cultural traditions. Indeed, Mediterranean lifestyle is a true *modus vivendi* that integrally promotes physical, mental, and social well-being. In addition, the Mediterranean diet stands out for its environmental sustainability because it is characterized mainly as a plant-based dietary pattern with low carbon and water footprints. Remarkably, Central Chile has a Mediterranean-like setting with plant and animal food production and availability patterns comparable to those present in countries located around the Mediterranean Sea. Therefore, this article reviews how promotion of Mediterranean lifestyle adherence in Chile offers great potential for management of the ongoing epidemiological transition to chronic diseases as well to promote psychological well-being within a unique food system and dietary sustainability vision for this Latin American country.

## 1. Introduction

Non-communicable diseases (NCDs) are major causes of morbidity and mortality, currently representing a large proportion of the overall economic healthcare burden worldwide [1], including in Chile. Moreover, cardiovascular, metabolic, and other chronic diseases are directly and strongly associated with poor mental health. Patients suffering from those chronic conditions report higher levels of depression [2,3], lower quality of life and decreased well-being [4]. Furthermore, predisposing psychosocial and lifestyle risk factors, such as an unhealthy diet and physical inactivity, are known to have driven this global increase in NCD prevalence and attributable deaths.

In Chile, 52% of total deaths occurred in 2011 were attributed to cardiovascular diseases (CVD) and cancer [5]. Furthermore, the 2016–2017 National Health Survey (NHS) showed high prevalence of obesity (31.2%), metabolic syndrome (MetS) (40.1%), diabetes (12.3%), and elevated CVD risk (26%) [6], with significant increases in obesity and diabetes compared to the 2003 NHS. On the other hand, depression and stress were detected in 15% and 30% of Chileans >15 years-old in 2016–2017, respectively [6], indicating a high prevalence of psychological dysfunction likely to negatively influence lifestyle choices as well as being linked with higher risk for chronic diseases [7]. Overall, the current epidemiological situation urgently calls for vision in design, implementation, and evaluation of nutritional interventions to attenuate the burden of NCDs in Chile.

Current dietary approaches essentially focus on providing only nutritional counseling, occasionally including support for dealing with psychological dysfunction or managing personal resources. However, a healthy lifestyle and effective behavioral change need to involve key positive psychological, interpersonal, and cultural resources as well as commitment to environmental sustainability, thereby promoting both individual and societal well-being. Indeed, dietary interventions may be more easily incorporated and followed by individuals if guided and supported by theory-based and contextually grounded psychological approaches in a comprehensive, positively-oriented social and environmental framework.

## 2. The Mediterranean Diet in the Current Chronic Disease Context

**Figure 1:**
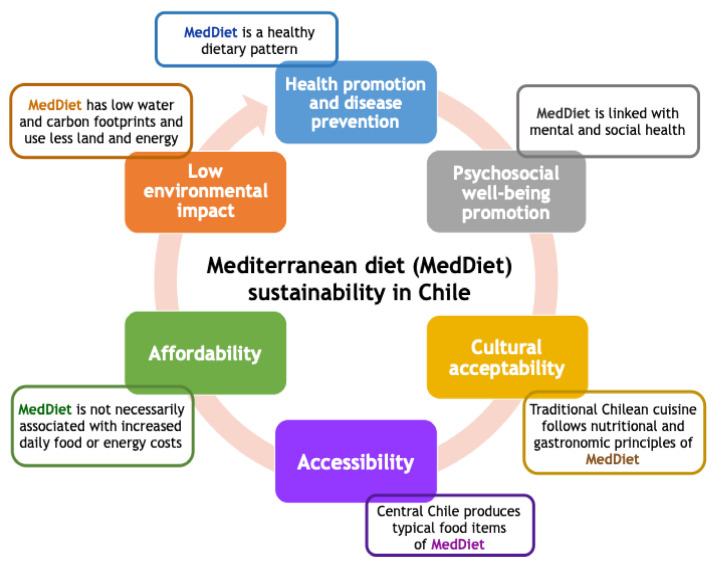
Mediterranean diet sustainability for promotion of physical, mental, and environmental health in Chile.

Currently, the Mediterranean diet (MedDiet) is considered one of the healthiest dietary patterns based on multiple observational studies [8,9,10,11,12,13] (Figure 1). In addition, intervention studies have evaluated the effect of MedDiets on long-term and hard clinical outcomes. One such trial, the secondary prevention Lyon Heart Study recruited individuals with previous myocardial infarction (MI) and randomly assigned them to a MedDiet enriched with α-linoleic acid, or a control group [14]. The MedDiet group exhibited a significantly lower recurrence of MI and reduced CVD mortality. On the other hand, PREDIMED (*Prevención con Dieta Mediterránea*) trial, a pioneer Spanish primary prevention study involving high cardiovascular risk participants, demonstrated that MedDiet, supplemented with olive oil or nuts, without caloric restriction or physical activity recommendation, reduced CVD events by 30% compared to a low-fat diet [15,16]. Further analyses in PREDIMED have shown that MedDiet reversed MetS and attenuated diabetes mellitus incidence [17], diabetic retinopathy [18], age-related cognitive decline [19], and invasive breast cancer occurrence [20]. Currently, PREDIMED-Plus is assessing the effect of a body weight-losing intensive lifestyle intervention based on energy-restricted Mediterranean diet, physical activity promotion, and behavioral support on primary prevention of CVD [21]. Interestingly, randomized clinical trials using MedDiet in non-Mediterranean countries have been recently reported in US [22] and Australia [23], although no such studies have yet been reported in Latin America.

The aim of this review is to summarize current evidence proposing that the Mediterranean lifestyle is a sustainable way to counteract the increasing incidence of chronic diseases, while promoting psychological well-being, thus highlighting the broad potential of this overall way of life in Chile.

## 3. Mediterranean-Type Ecosystem of Central Chile and Local Implications for Chronic Disease Management

Beyond the Mediterranean basin, four other world regions exhibit Mediterranean ecosystems, including Chile [24]. Indeed, Central Chile has a Mediterranean-like geographical setting with plant and animal food production and availability patterns comparable to those present in countries located around the Mediterranean Sea (Figure 1) [25]. Actually, ≈90% of annual food exports from Chile match a Mediterranean “food basket” [26]. Moreover, traditional Chilean cuisine uses ingredients and techniques that are very similar to those utilized in Southern Europe [27,28,29,30]. Therefore, promotion of MedDiet adherence using foods produced in Chile offers a great opportunity for management of the increasing prevalence of risk factors and NCDs in our country.

As discussed above, the benefits of MedDiet consumption have been well studied in Mediterranean countries and evaluated in cohorts from Northern Europe [31], US [32], and Australia [33]. However, until now, similar studies do not exist in Latin America. To overcome this void, application of locally adjusted and validated diet quality indexes—including MedDiet scores—is critical for identification of dietary correlations with chronic disease in specific populations. In this context, an adapted—incorporating local food patterns—self-applicable 14-point Chilean Mediterranean dietary index (Chilean MDI) was developed to evaluate MedDiet adherence and overall diet quality in the Chilean adult population [34]. A key finding was that only 10% of Chilean adults complied with an adequate MedDiet pattern showing diet-related demographic trends (i.e., adherence was higher in women, at older ages, and at higher educational levels) [34] that are consistent with those obtained with other MedDiet indexes in different populations.

Although overweight, obesity, and MetS are highly prevalent in Chile [6], there are very few large population-based studies investigating the link between dietary quality scores and these disease conditions. In this context, we published a report applying an abbreviated healthy diet index in the 2009–2010 Chilean National Health Survey [6]. Furthermore, using information collected in almost 25,000 Chileans from 2010 to 2015 through *Programa Aliméntate Sano* (www.alimentatesano.cl), an open-access online platform that provides lifestyle (including MedDiet) as well as health education, our MDI [34] showed that Chilean adults who exhibited higher adherence to MedDiet had lower prevalence of overweight, obesity and MetS [35]. Furthermore, this cross-sectional study [35] suggested that moderate improvement (by 2–3 points) in adherence to MedDiet measured by the 14-point Chilean MDI may be associated with significant decrease (by ≈10% absolute reduction) in obesity/overweight or MetS prevalence. However, this estimation requires further testing in a proper nutritional intervention trial.

In addition, Chilean children with high adherence to MedDiet showed lower blood pressure levels and better cardiovascular fitness [36,37], suggesting that early acquisition of this food intake pattern may be beneficial for cardiovascular physiology and prevention of relevant risk factors for NCD among young Latin Americans. Furthermore, high adherence to MedDiet was associated prospectively with reduced acute ischemic stroke severity in a Chilean hospital-register study [38]. Overall, there is notable opportunity for improvement in diet quality matching a MedDiet pattern with potential and significant impact on NCD risk in Chile.

So far, only preliminary studies, with various methodological pitfalls, have evaluated the actual impact of MedDiet interventions on some intermediate biomarkers, or limited clinical outcomes in Chilean subjects. In one small-size study, individuals enrolled to a three-month MedDiet intervention showed increased plasma antioxidant capacity and less oxidative damage [39], improved blood fatty acid profile [40], better hemostasis [41] and endothelial function [42] compared to those who ate a Western-type diet. Interestingly, moderate red wine consumption, included in the MedDiet intervention, improved antioxidant defense in both groups, counteracting oxidative damage caused by the Western diet [39,42]. In addition, uncontrolled calorie-restricted MedDiet decreased serum levels of end glycation products in premenopausal women who were overweight or obese [43]. More recently, a new Chilean randomized clinical trial has been registered to evaluate the effect of an avocado-supplemented MedDiet on lipid profile, particularly LDL cholesterol, in Chilean patients who are at high risk of recurrent ischemic stroke (ClinicalTrials.gov identifier NCT03524742). Finally, in a 12-month uncontrolled workplace intervention, aimed at Mediterranean style food availability at an on-site cafeteria combined with education promoting MedDiet-based meals, adherence of participants to MedDiet increased over time in association with a significant improvement in MetS components (e.g., abdominal obesity, hypertension, and low HDL cholesterol), while also correlating with an overall 35% reduction in MetS prevalence by the end of the study [44].

To obtain stronger evidence to support and more extensively implement this dietary pattern for NCD prevention and treatment in Chile, we are testing a locally adapted MedDiet pattern intervention—compared to a low fat diet alone—that is aimed to reverse MetS and to improve traditional and novel disease biomarkers in Chilean subjects diagnosed with this high risk condition for chronic diseases.

## 4. Beyond a Healthy Eating Pattern: Linking the Mediterranean Lifestyle to Mental Health and Well-Being

Consistent with the original Greek term δῐ́αιτᾰ/díaita, which means way of life, a Mediterranean lifestyle embraces a larger concept beyond mere food intake: it is, indeed, a comprehensive lifestyle that also promotes physical activity and appropriate rest/sleep along with important positive aspects derived from individual psychosocial features, community life, and culinary and cultural traditions that reinforce the sense of belonging and sharing in people living in Mediterranean countries (Figure 1) [45,46,47,48,49]. Thus, the Mediterranean lifestyle is a *modus vivendi* that integrally nurtures physical, mental, and social well-being.

Based in a mental disease framework, case-control and cross-sectional studies have supported an inverse association between baseline MedDiet adherence and depression (reviewed in [50,51,52,53,54]). With regard to intervention studies, the PREDIMED trial did not find a significant reduction in depression in the overall sample randomized to MedDiet, even though a significantly lower rate of depression was observed in diabetic patients allocated to MedDiet with supplementation of nuts [55]. Two additional trials (SMILES (Supporting the Modification of lifestyle In Lowered Emotional States), and MEAL (Mediterranean healthy Eating, Aging and Lifestyle)) are evaluating the impact of implementing a MedDiet pattern on depression [56,57].

Moving toward a positive perspective, we conceptualize mental and psychosocial well-being [58] as encompassing three components: a state of emotional/subjective well-being [59] together with effective functioning of the individual (psychological well-being (PWB)) [60] as part of a positive community (social well-being) [61]. First, emotional, subjective or hedonic well-being involves an emotional pattern defined by high positive/negative affect ratio, happiness, and life satisfaction [59,62,63]. Secondly, psychological or eudaimonic well-being includes self-acceptance, autonomy, positive relationships with others, environmental mastery, personal growth, and purpose in life [60,64,65]. Finally, social well-being comprises community integration, acceptance, contribution, actualization, and coherence [61]. These well-being models were further integrated in the positive Mental Health Continuum (MHC) [66].

In particular, the PWB model [64,65] focuses on under-appreciated aspects of affirmative psychological functioning that have been strongly linked to different positive social, behavioral, biological, and health parameters and outcomes (reviewed in [64,65,67]). Indeed, various features of eudaimonic well-being are associated with healthier behaviors (e.g., exercise, good sleep, weight control) and better physiological regulation and reduced inflammatory and CVD risk factors (reviewed in [65,67]). For instance, persistently high eudaimonic PWB predicts better HDL cholesterol and triglyceride levels in the longitudinal MIDUS (Midlife Development in United States) cohort [68], and it is also cross sectionally associated with lower levels of circulating ceramides, a lipid class involved in metabolic diseases and aging [Berkowitz et al., under revision]. Particular interest has centered on ”purpose in life”, a key dimension of eudaimonic well-being, and its links to healthier habits, more preventive behavior, decreased cognition impairment, reduced Alzheimer and CVD incidence, low total mortality, and longevity (reviewed in [69]).

Based in its broader conceptual paradigm and its implications for promotion of preventive behaviors, the psychological well-being model fits impressively with the larger concept of a Mediterranean lifestyle, including additional and reciprocal benefits on physical, mental, and social health. Considering the positive sociocultural and psychological context in which MedDiets have evolved, it may be particularly useful to explore whether adherence to this lifestyle pattern is linked with various positively operationalized dimensions of psychosocial well-being. MedDiet intake/adherence has been correlated with better health-related quality of life in some cross-sectional or longitudinal cohorts from Greece, Spain and Italy [70,71,72,73,74,75,76,77] as well in US [78], but these findings were not reproduced in other populations [79,80]. More detailed studies, appropriately evaluating a reciprocal link between intake of MedDiets and using more comprehensive measures of subjective, eudaimonic and social well-being are needed.

In this regard, longitudinal evidence demonstrated that baseline adherence to MedDiet predicts better psychological vitality [74] and emotional response via increasing positive affect [75], hinting that improving MedDiet-based food habits may bring increased well-being. Interestingly, adherence to MedDiet correlated cross-sectionally with self-esteem and self-regulation in Chilean children [81,82]. Furthermore, using a positive mental health questionnaire [66] in a cross sectional internet survey, Chilean adults reporting a healthy eating pattern according to the Chilean MDI [34] showed increased prevalence of high levels of PWB as well as increased optimism, and higher subjective vitality [major preliminary findings summarized in ref. [30] and Echeverría et al., manuscript in preparation]. More recently, we found that a Mediterranean-like healthy eating pattern correlated inversely with MetS prevalence and positively with PWB scores in MIDUS [Echeverría et al., unpublished data]. Taken together, these findings suggest an association between high MedDiet adherence and better psychological well-being within as well as outside the Mediterranean basin.

If the associations between MedDiet and well-being are reciprocally causative, improvement in lifestyle habits based on Mediterranean patterns may bring increased well-being, and alternatively, promotion of various aspects constituting PWB may facilitate acquisition and routine practice of a healthy MedDiet pattern, thus generating a salutogenic cycle that leads to reduced risk of physical and psychological chronic diseases and improved overall positive health.

Promotion of well-being per se is, indeed, an emerging goal in mental healthcare, shifting the focus from merely treating or preventing mental disease to actively enhancing positive aspects of mental health. Moreover, an increasing number of intervention approaches, including well-being therapy [83,84,85,86,87], have shown increased subjective well-being and enhanced positive psychological functioning in clinical and non-clinical study samples [88,89,90]. Moreover, meta-analyses of randomized trials have revealed that these interventions improved various aspects of psychological well-being with moderate effect sizes across studies and significant impact on various categories of outcome variables [88,89,90]. More recently, randomized controlled trials based on positive affect and mood-related interventions have led to improvements in healthy behaviors (medication, diet, physical activity, and exercise adherence) [91,92,93,94,95,96]. If PWB interventions are feasible and effective in boosting adherence to key healthy behaviors, they may also be a novel cost-effective tool to improve biomarkers as well as functional and medical outcomes related to chronic disease conditions. In fact, we are currently testing a PWB-based intervention as an innovative approach to increase MedDiet initiation and adherence when managing MetS in Chilean subjects, promoting the broader concept of Mediterranean lifestyle, not exclusively focused on a nutritional point of view.

## 5. Sustainability of the Mediterranean Diet

A critical target in any future vision about food intake is that of a sustainable healthy diet that is health-promoting and has a low environmental impact involving food that is accessible, affordable, and culturally endorsed. The combination of all these objectives embodies an overarching commitment to prevent malnutrition in all its forms, thereby reducing dietary-related NCDs, while simultaneously preserving biodiversity and planetary health [97].

This is the current context: due to rising contributions (up to 37%) of global greenhouse gases (GHG) emissions worldwide, including 10% derived from food waste and losses [98], dietary patterns and food systems become a critical pillar of climate change mitigation. Overall, plant-based foods have a lower environmental influence, while animal-based and ultra-processed foods have a higher environmental impact [99]. The fact that healthy diets are more environmentally sustainable does not necessarily mean that every healthy diet is environmentally sustainable, nor that every environmentally sustainable diet is healthy [100]. As health benefits have been discussed above, this section focuses on other dimensions—namely, environmental and cultural issues together with physical and economical access—of Mediterranean food patterns as sustainable diets.

### 5.1. Mediterranean Diet and the Environment

**Figure 2:**
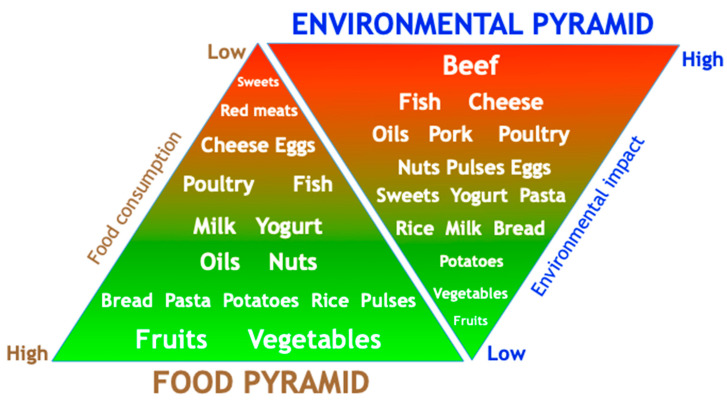
The double food and environmental pyramid of the Mediterranean diet (adapted from [105]).

In comparison with a standard Western diet, consumption of Mediterranean food patterns in adults has lower water and carbon footprints (WF and CF, respectively) and use less land and energy [100], see also [101] and [102] in this Special Issue (Figure 2). Even though MedDiet considers a higher intake of oils and nuts compared to Western diet, the associated WF impact of these food items is counteracted by its lower intake of animal products [103]. Similarly, legumes are an important protein source in MedDiets, a food group that has a very little CF compared with beef, the most common protein source in Western diets [104]. On the other hand, animal-based dairy intake as part of a MedDiet has high CF, but it is still lower than other highly consumed beverages, such as soda [104]. Moreover, MedDiet may lead to even better average environmental outcomes in comparison with a high-fat vegan diet, which includes lofty consumption of oil and nuts with great WF, of which nuts are 74% produced under blue water stress [103]. Even though vegan diets are usually the most environmentally sustainable, in some cases, land well-suited for crop production [100] can be retrieved and used for animal grazing linked to beef and dairy production, optimizing resources and supporting thousands of livelihoods with a somewhat positive economically sustainable impact. Thus, various comprehensive synergies and trade-offs need to be identified, assessed, and taken into consideration when recommending a sustainable diet.

Normally, MedDiet also includes locally produced foods, which lower transportation-related CF [106]. Furthermore, Mediterranean farming is seen as a protective model for family agriculture and a model of sustainable rural development [106]. Despite the locally preferred food production and consumption, there are situations where production methods themselves may be critical, thus sometimes locally grown produce may have a detrimental CF effect [107]. In addition, local data should be assessed, and alternatives carefully weighed when making diet sustainability recommendations.

From an overall budgetary perspective, intrinsic environmental costs associated with unhealthy diets are foreseen to reach USD 1.7 trillion by 2030, even higher than their own sanitary associated costs (USD 1.3 trillion) [108]. In addition, obesity is not only a matter of economic and public health burden, but represents an environmental issue in its own right. An overweight person eats almost 20% more calories from food, translating into more resource consumption, biodiversity loss, and additional GHG emissions [109,110]. Thus, implementing MedDiets as a healthy food pattern has important implications related to links between obesity and environmental and economic sustainability.

Overall, the MedDiet is a well-studied dietary pattern that is both healthy and environmentally sustainable. In fact, the Barilla Center for Food and Nutrition Foundation (BCFN) has proposed the Double Pyramid of MedDiet, which includes its healthy food pattern as well as an upside-down environmental-pyramid (Figure 2) [105]. Furthermore, the Food and Agriculture Organization (FAO) of the United Nations has acknowledged the MedDiet as an example of a sustainable diet because it is focused mainly on plant-based food and local produce [97,100,111].

### 5.2. Mediterranean Diet and Cultural Acceptance

Even though MedDiet is a sustainable healthy diet, many people are unlikely to adhere to a food pattern if it is not culturally acceptable. That said, cultural issues should not be a limitation for transferability of this dietary style beyond the Mediterranean basin. MedDiets, including their food components and culinary methods, are not a strict, but indeed constitute a flexible, dietary pattern that can be locally adapted based on food availability and cuisine traditions [48]. However, in the best of all worlds, this cultural adaptation should preserve key dimensions of sustainable diets: accessibility, affordability as well as human and planetary health.

Remarkably, MedDiets are known for their care regarding food ingredients and preparation together with socialization, both of which underscore cultural values [111,112]. Indeed, a Mediterranean-like diet should include the traditional foods of native cultures from other Mediterranean world regions, as happens in Central Chile. Chilean culture around food is strongly shaped by geographic localization and its local food traditions, which originated from indigenous people as well as Southern Europe. It is also characterized as a way to show appreciation and care to others and it plays a central role on many traditional celebrations.

In Chile, over 82% of 400 surveyed people reported that cooking is a pleasure for them, most of them cook with a companion, which contributes to social continuity, and they favor (70%) Chilean gastronomy [113]. Interestingly, the MedDiet has already been translated into specific culturally adapted food and preparations in other countries [33]. Chile strongly fits in this dietary pattern within its traditional dishes. For instance, chestnuts and pinon nuts are important in the Chilean food culture and, as groundnuts, they have less WF than nuts from trees [103]. In addition, avocados, which are not native to the Mediterranean basin, but are produced and highly consumed in Chile, are a great addition to a MedDiet as fresh local produce with a high monounsaturated fatty acid content, somewhat mimicking olive oil composition [29,30]. Finally, many traditional Chilean food preparations use a Spanish and Italian-like sofrito, a mixture of lightly fried onions and garlic, usually with tomatoes and other vegetables, as a base for soups and stews [29,30].

Limited time available for cooking may be one of the hurdles to adoption of more healthy diets. When considering MedDiets, cooking time within a particular food preparation culture is also a critical factor helping to shift toward eating fresh, plant-based foods and staying away from prepackaged meals or on restaurants. Since MedDiet involves significant home food preparation, time scarcity may be an important barrier to its implementation, particularly at low socioeconomic levels. Furthermore, cooking has been historically assigned to women, and there is an overall trend to a reduction in this household activity as women increasingly work outside the home [114]. This situation thus could be a potential barrier to change food habits and is an issue needing attention. Indeed, home cooking and family meals are a fundamental structure of healthy diets, and time restriction carries a huge risk of dietary changes.

Even though Chile has a strong potential in the cultural adoption of MedDiet because of food availability, preferences and habits (Figure 1), further promotion of this dietary pattern should be modeled by local traditions through nutritional and sociocultural surveys in order to identify similarities and differences relative to MedDiets and adapt local habits without changing our local food and eating culture entirely [48,107,114].

### 5.3. Mediterranean Diet and Economical Costs and Accessibility

For most consumers, price and taste are the main influences on decisions about what food to buy and eat [115,116]. Whether the MedDiet is affordable has been debated, and the evidence thus far is inconclusive [115], which is the same scenario for most sustainable diets [100]. Feasibility and cost-effectiveness analyses, involving expertise well beyond the biomedical field, will be required to further establish specific dietary patterns as general public policy seeking to promote health and quality of life and prevent the current NCD burden in our population.

Several studies show that economic constraints determine consumption of unhealthy diets characterized by high energy density and palatability [115]. In contrast, high adherence to a healthy dietary pattern is usually associated with higher expenses [108]. In Australia, a sustainable and healthy diet was 30% more expensive than the usual diet for those economically vulnerable [100]. Furthermore, the latest report of the State of Food Insecurity in the World [109] shows that a healthy diet costs up to 5 times more than energy sufficient diets, and Latin America and the Caribbean is the most expensive region (34% higher cost than the global average) for buying food. In this context, a quality food basket was 32% more expensive compared to a basic food basket in Chile, thus precluding 27% of the population from its access [117].

Nonetheless, it is debatable whether the MedDiet is more expensive [115,118,119]. With regard to absolute costs, MedDiet based on common Greek dietary choices varied widely from 5.4 to 83.6 €/week in men and from 10.9 to 55.5 €/week in women with on overall average of 25–26 €/week in both sexes [120]. Two studies in Spanish subjects showed that MedDiet pattern was more expensive than a Westernized dietary pattern or an unhealthy diet after adjusting for potential confounders [121,122]. Another study corroborated that MedDiet foods can be obtained at very broad price ranges (per 100 g or 1000 kcal) and identified some conditions to reach affordable MedDiet costs: high intake of grains, legumes, nuts, vegetables and fruits and lower consumption of leafy greens and fresh fish [119]. Furthermore, an adapted version of MedDiet for low income subjects in US led to increased cost due to higher intake of vegetables, fruits, legumes, nuts and seeds, canola/olive oil, whole grains, poultry and fish, but reduced expenses from less red meat, refined grains, desserts and sweets, and fast food [123]. The authors concluded that predefined MedDiet-oriented choices are not necessarily associated with increased overall daily dietary cost or energy costs, thus economics should not be construed a priori as a barrier to initiation and adherence to MedDiet [123]. However, there are significant challenges when estimating food costs: price of similar food items can vary considerably depending on various factors, such as volume/weight, quality, brand, site of purchase among others. Indeed, trade-offs and cost-effectiveness should be taken into consideration. In addition, United States Department of Agriculture (USDA) analyses have shown that those who cooked at home more frequently had healthier diets and spent less money overall [100]. In the same direction, a more sustainable diet could be achieved while keeping costs low by eating less calories and more plant-based foods [100].

Furthermore, actual cost-effectiveness of nutritional interventions is relevant to support the decisions of different stakeholders, such as clinicians, health care managers, and policy-makers. Recent estimations show Chileans will decrease their life expectancy by 3.5 years over the next three decades, and that obesity-related conditions could reduce gross domestic product of the country by almost 4%, and concomitantly, cost almost 9% of the nation health budget [124]. Nonetheless, there are limited cost-effectiveness analyses of nutrition interventions [115]. From this perspective, MedDiet appears to be cost-effective based on QALY (quality adjusted life year) assessment using good quality trials in comparison with other interventions [125,126]. In particular, data from the Lyon Diet Heart Study combined with conservative assumptions indicate that MedDiet is cost-effective in a high-risk population after a first myocardial infarction with a very favorable return on investment [125]. In addition, total healthcare cost in subjects without prior atherosclerotic CVD was estimated to be 10-fold higher in those who showed low adherence to MedDiet compared to subjects those who were closer to this diet pattern [127]. Then, economic evaluation should be increasingly applied to ensure that scarce resources are allocated more efficiently to reduce the NCD burden through implementation of a healthy dietary pattern.

However, food intake-related economic issues differ between countries and their populations. Some evidence suggests a low cost predefined MedDiet may be feasible for low-income subjects within US [119]. Nevertheless, it is not clear whether regions outside the Mediterranean basin, such as Chile, can indeed adopt and maintain long-term dietary behaviors consistently with a MedDiet pattern from an economic perspective. Thus, costs and budgetary issues must be considered and addressed when designing and implementing a MedDiet-based interventional approach beyond the Mediterranean Sea.

On the other hand, physical accessibility of food is a critical point for adoption of eating habit changes. Some countries in northern Europe are increasingly adopting the MedDiet due to greater availability of fruits and vegetables in local stores [48]. Indirectly, household income also affects access to healthy foods by concentrating them in higher socioeconomic neighborhoods [114]. In contrast, farmers’ market in Chile are commonly located in more vulnerable areas, they supply fruits and vegetables for 70% of the country population and are up to 30% cheaper than supermarkets [128], thus facilitating accessibility and affordability these food groups.

Generally speaking, cost and accessibility for application of a Mediterranean-type food pattern in Chile may not be necessarily prohibitive (Figure 1) and can be adjusted, according to the availability of resources, through greater use of cheaper food items (e.g., legumes, local nuts, grains, canola oil), moderate—rather than high increase of olive oil intake, reduced consumption of more expensive foods (e.g., red meats). In fact, a preliminary analysis of the uncontrolled intervention study previously conducted by our group [44] show no significant cost increase at the workplace cafeteria in which a shift towards Mediterranean style on food was implemented [Echeverría G et al., unpublished results].

If healthy diets are more expensive than unhealthy diets, human and planetary health will not be achieved through mere education and intention. Indeed, educational efforts on healthy food patterns have already been implemented in Chile, such as *5 al Día* (www.5aldia.cl), *Aliméntate Sano* (www.alimentatesano.cl), and *Elige Vivir Sano* (www.eligevivirsano.gob.cl) programs. However, their impact on consumption of locally available foods and health education has not been evaluated in public discourse or reported in scientific literature.

Despite of well-intentioned attempts, education is not commonly enough to effectively change eating habits as a consequence of economic restrictions. However, costs should not remain a central obstacle and insuperable barrier against adoption of MedDiet or any healthy diet. Of critical importance are public food policies such as fiscal taxing, subsidies, and planning that encourage tackling nutrition, health and environmental inequities as linked objectives. Indeed, studies on long term benefits of MedDiet or other plant-based food patterns on national health-related costs should help in prompting policies to subsidize growing healthier foods and deterring meat-based diets among overall populations.

Finally, we must be aware of all issues discussed in this section and address them appropriately because of the worrisome trend to lower MedDiet adherence detected from 1960s to early 21st century worldwide [129], including southern Mediterranean countries [130], even though some stabilization and improvement seems to be occurring lately [129]. Thus, whole-food plant based diets, in which MedDiet fits nicely, should be strongly encouraged based on current scientific evidence as indicated by the 2019 Lancet Eat Commission Report aimed to nurture human and planetary health [131].

## 6. Conclusions

Even though it may not be the only approach to oppose the alarming increase in obesity and associated chronic disease risk and incidence, MedDiet may improve health and quality of life in the Chilean population. Evidence supporting health benefits of MedDiet, its relationship with PWB and its environmental impact in Chile (Figure 1) is still preliminary and limited; however, it is encouraging to show emerging findings and underscore the longer-term potential. Thus, additional intervention studies using a locally adapted and feasible MedDiet intervention, context-tailored support to behavior change based on the psychological well-being theory, concomitant sustainability analyses, and more comprehensive outcome evaluations are required—as an in situ proof of concept—to further emphasize and more extensively implement this dietary pattern for prevention and treatment of chronic diseases in our population.

## References

[1] WHO (2014). Global Status Report on Noncommunicable Diseases. Attaining the Nine Global Noncommunicable Diseases Targets: A Shared Responsibility.

[2] Carney R.M., Freedland K.E. (2017). Depression and coronary heart disease. Nat. Rev. Cardiol..

[3] Pan A., Keum N., Okereke O.I., Sun Q., Kivimaki M., Rubin R.R., Hu F.B. (2012). Bidirectional Association Between Depression and Metabolic Syndrome: A systematic review and meta-analysis of epidemiological studies. Diabetes Care.

[4] DuBois C.M. (2015). Relationships between positive psychological constructs and health outcomes in patients with cardiovascular disease: A systematic review. Int. J. Cardiol..

[5] MINSAL Chile 2018. http://www.deis.cl/defunciones-y-mortalidad-por-causas.

[6] MINSAL Chile 2018. https://www.minsal.cl/wp-content/uploads/2017/11/ENS-2016-17_PRIMEROS-RESULTADOS.pdf.

[7] Pedersen S.S., Von Känel R., Tully P.J., Denollet J. (2017). Psychosocial perspectives in cardiovascular disease. Eur. J. Prev. Cardiol..

[8] García-Fernández E., Rico-Cabanas L., Rosgaard N., Estruch R., Bach-Faig A. (2014). Mediterranean Diet and Cardiodiabesity: A Review. Nutrients.

[9] Dussaillant C., Echeverría G., Urquiaga I., Velasco N., Rigotti A. (2016). Current evidence on health benefits of the Mediterranean diet. Rev. Med. Chile.

[10] Dinu M., Pagliai G., Casini A., Sofi F. (2017). Mediterranean diet and multiple health outcomes: An umbrella review of meta-analyses of observational studies and randomised trials. Eur. J. Clin. Nutr..

[11] Soltani S., Jayedi A., Shab-Bidar S., Becerra-Tomas N., Salas-Salvadó J. (2019). Adherence to the Mediterranean Diet in Relation to All-Cause Mortality: A Systematic Review and Dose-Response Meta-Analysis of Prospective Cohort Studies. Adv. Nutr..

[12] Kargın D., Tomaino L., Serra-Majem L. (2019). Experimental Outcomes of the Mediterranean Diet: Lessons Learned from the Predimed Randomized Controlled Trial. Nutrients.

[13] Sánchez-Sánchez M.L., García-Vigara A., Hidalgo-Mora J.J., García-Pérez M.Á., Tarín J., Cano A. (2020). Mediterranean diet and health: A systematic review of epidemiological studies and intervention trials. Maturitas.

[14] De Lorgeril M., Renaud S., Salen P., Monjaud I., Mamelle N., Martin J., Guidollet J., Touboul P., Delaye J. (1994). Mediterranean alpha-linolenic acid-rich diet in secondary prevention of coronary heart disease. Lancet.

[15] Estruch R., Ros E., Salas-Salvadó J., Covas M.-I., Corella D., Arós F., Gómez-Gracia E., Ruiz-Gutiérrez V., Fiol M., Lapetra J. (2013). Primary Prevention of Cardiovascular Disease with a Mediterranean Diet. N. Engl. J. Med..

[16] Estruch R., Ros E., Salas-Salvadó J., Covas M.I., Corella D., Arós F., Gómez-Gracia E., Ruiz-Gutiérrez V., Fiol M., Lapetra J. (2018). Retraction and Republication: Primary Prevention of Cardiovascular Disease with a Med-iterranean Diet. N. Engl. J. Med..

[17] Salas-Salvadó J., Bulló M., Babio N., Martínez-González M.Á., Ibarrola-Jurado N., Basora J., Estruch R., Covas M.I., Corella D., Arós F. (2010). Reduction in the Incidence of Type 2 Diabetes With the Mediterranean Diet: Results of the PREDIMED-Reus nutrition intervention randomized trial. Diabetes Care.

[18] Diaz-Lopez A. (2015). Mediterranean diet, retinopathy, nephropathy, and microvascular diabetes complications: A post hoc analysis of a randomized trial. Diabetes Care.

[19] Valls-Pedret C., Sala-Vila A., Serra-Mir M., Corella D., de la Torre R., Martínez-González M.Á., Martínez-Lapiscina E.H., Fitó M., Pérez-Heras A., Salas-Salvadó J. (2015). Mediterranean Diet and Age-Related Cognitive Decline. JAMA Intern. Med..

[20] Toledo E., Salas-Salvadó J., Donat-Vargas C., Cosiales P.B., Estruch R., Ros E., Corella D., Fitó M., Hu F.B., Arós F. (2015). Mediterranean Diet and Invasive Breast Cancer Risk Among Women at High Cardiovascular Risk in the PREDIMED Trial. JAMA Intern. Med..

[21] Martínez-González M., Buil-Cosiales P., Corella D., Bulló M., Fitó M., Vioque J., Romaguera D., Martínez J.A., Wärnberg J., López-Miranda J. (2018). Cohort Profile: Design and methods of the PREDIMED-Plus randomized trial. Int. J. Epidemiol..

[22] Sotos-Prieto M. (2017). Rationale and design of feeding America’s bravest: Mediterranean diet-based intervention to change firefighters’ eating habits and improve cardiovascular risk profiles. Contemp. Clin. Trials.

[23] Itsiopoulos C., Kucianski T., Mayr H.L., Van Gaal W.J., Martínez-González M.Á., Vally H., Kingsley M., Kouris-Blazos A., Radcliffe J., Segal L. (2018). The AUStralian MEDiterranean Diet Heart Trial (AUSMED Heart Trial): A randomized clinical trial in secondary prevention of coronary heart disease in a multiethnic Australian population: Study protocol. Am. Heart J..

[24] Köppen V., Köppen V., Geiger‎ R. (1936). Das Geographische System der Climate.

[25] Armesto J.J. (2007). The Mediterranean environment of Central Chile.

[26] Chilealimentos Evolución Exportaciones de Alimentos. https://chilealimentos.com/ventajas_categoria/evolucion-exportaciones-de-alimentos/.

[27] Rozowski J. (2004). Is the Chilean diet a Mediterranean-type diet?. Biol. Res..

[28] Urquiaga I., Echeverría G., Dussaillant C., Rigotti A. (2017). Origin, components and mechanisms of action of the Mediterranean diet. Rev. Med. Chile.

[29] Echeverría G., Dussaillant C., McGee E., Inés U., Velasco N., Rigotti A. (2017). Mediterranean diet beyond the mediterranean basin: Chronic disease prevention and treatment. Mediterranean Identities—Environment, Society, Culture.

[30] Echeverría G., Dussaillant C., McGee E.E., Mena C., Nitsche M.P., Inés U., Bitran M., Pedrals N., Rigotti A. (2018). Promoting and Implementing the Mediterranean Diet in the Southern Hemisphere: The Chilean Experience. Eur. J. Clin. Nutr..

[31] Panico S., Mattiello A., Panico C., Chiodini P. (2013). Mediterranean Dietary Pattern and Chronic Diseases. Cancer Treat. Res..

[32] Harmon B., Boushey C.J., Shvetsov Y.B., Ettienne R., Reedy J., Wilkens L.R., Le Marchand L., Henderson B., Kolonel L.N. (2015). Associations of key diet-quality indexes with mortality in the Multiethnic Cohort: The Dietary Patterns Methods Project. Am. J. Clin. Nutr..

[33] Mantzioris E., Villani A. (2019). Translation of a Mediterranean-Style Diet into the Australian Dietary Guidelines: A Nutritional, Ecological and Environmental Perspective. Nutrients.

[34] Echeverria G., Urquiaga I., Concha M.J., Dussaillant C., Villarroel L., Velasco N., Leighton F., Rigotti A. (2016). Validation of self-applicable questionnaire for a Mediterranean dietary index in Chile. Rev. Med. Chile.

[35] Echeverría G., McGee E.E., Urquiaga I., Jiménez P., D’Acuña S., Villarroel L., Velasco N., Leighton F., Rigotti A. (2017). Inverse Associations between a Locally Validated Mediterranean Diet Index, Overweight/Obesity, and Metabolic Syndrome in Chilean Adults. Nutrients.

[36] Garcia-Hermoso A., Vegas-Heredia E.D., Fernández-Vergara O., Ceballos-Ceballos R., Andrade-Schnettler R., Arellano-Ruiz P., Ramírez-Vélez R. (2019). Independent and combined effects of handgrip strength and adherence to a Mediterranean diet on blood pressure in Chilean children. Nutrients.

[37] Delgado-Floody P., Alvarez C., Caamaño-Navarrete F., Jerez-Mayorga D., Latorre-Román P. (2020). Influence of Mediterranean diet adherence, physical activity patterns, and weight status on cardiovascular response to cardiorespiratory fitness test in Chilean school children. Nutrients.

[38] Lavados P.M., Mazzon E., Rojo A., Brunser A.M., Olavarría V.V. (2020). Pre-stroke adherence to a Mediterranean diet pattern is associated with lower acute ischemic stroke severity: A cross-sectional analysis of a prospective hospital-register study. BMC Neurol..

[39] Urquiaga I., Strobel P., Perez D., Martinez C., Cuevas A., Castillo O., Marshall G., Rozowski J., Leighton F. (2010). Mediterranean diet and red wine protect against oxidative damage in young volunteers. Atherosclerosis.

[40] Urquiaga I., Guasch V., Marshall G., San Martín A., Castillo O., Rozowski J., Leighton F. (2004). Effect of Mediterranean and Occidental diets, and red wine, on plasma fatty acids in humans. An intervention study. Biol. Res..

[41] Mezzano D., Leighton F., Strobel P., Martínez C., Marshall G., Cuevas A., Castillo O., Panes O., Muñoz B., Rozowski J. (2001). Complementary effects of Mediterranean diet and moderate red wine intake on haemostatic cardiovascular risk factors. Eur. J. Clin. Nutr..

[42] Leighton F., Cuevas A., Guasch V., Pérez D.D., Strobel P., Martín A.S., Urzua U., Díez M.S., Foncea R., Castillo O. (1999). Plasma polyphenols and antioxidants, oxidative DNA damage and endothelial function in a diet and wine intervention study in humans. Drugs Under Exp. Clin. Res..

[43] Rodriguez J.M. (2015). Reduction of serum advanced glycation end-products with a low calorie Mediterra-nean diet. Nutr. Hosp..

[44] Leighton F., Polic G., Strobel P., Perez D., Martinez C., Vásquez L., Castillo O., Villarroel L., Echeverría G., Inés U. (2009). Health impact of Mediterranean diets in food at work. Public Health Nutr..

[45] Willett W.C., Sacks F., Trichopoulou A., Drescher G., Ferro-Luzzi A., Helsing E., Trichopoulos D. (1995). Mediterranean diet pyramid: A cultural model for healthy eating. Am. J. Clin. Nutr..

[46] Trichopoulou A., Lagiou P. (2009). Healthy Traditional Mediterranean Diet: An Expression of Culture, History, and Lifestyle. Nutr. Rev..

[47] Bach-Faig A., Berry E.M., Lairon D., Reguant J., Trichopoulou A., Dernini S., Medina F.X., Battino M., Belahsen R., Miranda G. (2011). Mediterranean Diet Foundation Expert Group. Mediterranean diet pyramid today. Science and cultural updates. Public Health Nutr..

[48] Lăcătușu C.-M., Grigorescu E.-D., Floria M., Onofriescu A., Mihai B.-M. (2019). The Mediterranean Diet: From an Environment-Driven Food Culture to an Emerging Medical Prescription. Int. J. Environ. Res. Public Health.

[49] UNESCO Mediterranean Diet: UNESCO Intangible Cultural Heritage. https://ich.unesco.org/es/RL/la-dieta-mediterranea-00884.

[50] Psaltopoulou T., Sergentanis T.N., Panagiotakos D.B., Sergentanis I.N., Kosti R., Scarmeas N. (2013). Mediterranean diet, stroke, cognitive impairment, and depression: A meta-analysis. Ann. Neurol..

[51] Martínez-González M.A., Sánchez-Villegas A. (2016). Food patterns and the prevention of depression. Proc. Nutr. Soc..

[52] Molendijk M., Molero P., Sánchez-Pedreño F.O., van der Does W., Martínez-González M.A. (2018). Diet quality and depression risk: A systematic review and dose-response meta-analysis of prospective studies. J. Affect. Disord..

[53] Lassale C., Batty G.D., Akbaraly T. (2019). Reply to Veronese and Smith: Healthy dietary indices and risk of depressive outcomes: A systematic review and meta-analysis of observational studies. Mol. Psychiatry.

[54] Shafiei F., Salari-Moghaddam A., Larijani B., Esmaillzadeh A. (2019). Adherence to the Mediterranean diet and risk of depression: A systematic review and updated meta-analysis of observational studies. Nutr. Rev..

[55] Sanchez-Villegas D.A., Martinez-Gonzalez M.A., Estruch R., Salas-Salvadó J., Corella D., Covas M.-I., Arós F., Romaguera D., Gómez-Gracia E., Lapetra J. (2013). Mediterranean dietary pattern and depression: The predimed randomized trial. BMC Med..

[56] Opie R.S., O’Neil A., Jacka F.N., Pizzinga J., Itsiopoulos C. (2017). A modified Mediterranean dietary intervention for adults with major depression: Dietary protocol and feasibility data from the SMILES trial. Nutr. Neurosci..

[57] Grosso G., Marventano S., D’Urso M., Mistretta A., Galvano F. (2016). The Mediterranean healthy eating, ageing, and lifestyle (MEAL) study: Rationale and study design. Int. J. Food Sci. Nutr..

[58] World Health Organization (2005). Promoting Mental Health. Concepts. Emerging Evidence.

[59] Diener E. (1984). Subjective well-being. Psychol. Bull..

[60] Ryff C.D. (1989). Happiness is everything, or is it? Explorations on the meaning of psychological well-being. J. Pers. Soc. Psychol..

[61] Shapiro A., Keyes C.L.M. (2008). Marital Status and Social Well-Being: Are the Married Always Better Off?. Soc. Indic. Res..

[62] Diener E., Lucas R.E., Oishi S. (2018). Advances and Open Questions in the Science of Subjective Well-Being. Collabra Psychol..

[63] Diener E., Oishi S., Tay L. (2018). Advances in subjective well-being research. Nat. Hum. Behav..

[64] Ryff C.D. (2008). Know yourself and become who you are: A eudaimonic approach to well-being. J. Happiness Stud..

[65] Ryff C.D. (2014). Psychological Well-Being Revisited: Advances in the Science and Practice of Eudaimonia. Psychother. Psychosom..

[66] Keyes C.L.M. (2002). The Mental Health Continuum: From Languishing to Flourishing in Life. J. Health Soc. Behav..

[67] Ryff C.D., Boylan J.M., Kirsch J.A., Lee M.T., Kubzansky L.D., VanderWeele T.J. (2021). Eudaimonic and Hedonic Well-Being: An Integrative Perspective with Linkages to Socio-demographic Factors and Health. Measuring Well-being: Interdisciplinary Perspectives from the Social Sciences and the Humanities.

[68] Radler B.T., Rigotti A., Ryff C.D. (2018). Persistently high psychological well-being predicts better HDL cholesterol and triglyceride levels: Findings from the midlife in the U.S. (MIDUS) longitudinal study. Lipids Health Dis..

[69] Ryff C.D. (2017). The Benefits of Purposeful Life Engagement on Later-Life Physical Function. JAMA Psychiatry.

[70] Munoz M.A. (2009). Regicor and hermes investigators. Adherence to the Mediterranean diet is associated with better mental and physical health. Br. J. Nutr..

[71] Costarelli V., Koretsi E., Georgitsogianni E. (2013). Health-related quality of life of Greek adolescents: The role of the Mediterranean diet. Qual. Life Res..

[72] Grao-Cruces A., Nuviala A., Fernández-Martínez A., Porcel-Gálvez A.M., Moral-García J.E., Martínez-López E.-J. (2013). Adherence to the Mediterranean diet in rural and urban adolescents of southern Spain, life satisfaction, anthropometry, and physical and sedentary activities. Nutr. Hosp..

[73] Bonaccio M., Di Castelnuovo A., Bonanni A., Costanzo S., de Lucia F., Pounis G., Zito F., Donati M.B., de Gaetano G., Iacoviello L. (2013). Adherence to a Mediterranean diet is associated with a better health-related quality of life: A possible role of high dietary antioxidant content. BMJ Open.

[74] Sánchez P.H., Ruano C., De Irala J., Ruiz-Canela M., Martínez-González M.A., Sánchez-Villegas A. (2012). Adherence to the Mediterranean diet and quality of life in the SUN Project. Eur. J. Clin. Nutr..

[75] Holt M.E., Lee J.W., Morton K.R., Tonstad S. (2014). Mediterranean diet and emotion regulation. Mediterr. J. Nutr. Metab..

[76] Galilea-Zabalza I., Buil-Cosiales P., Salas-Salvadó J., Toledo E., Ortega-Azorín C., Díez-Espino J., Vázquez-Ruiz Z., Zomeño M.D., Vioque J., Martínez J.A. (2018). Mediterranean diet and quality of life: Baseline cross-sectional analysis of the predimed-plus trial. PLoS ONE.

[77] Bonaccio M., Di Castelnuovo A., Costanzo S., Pounis G., Persichillo M., Cerletti C., Donati M.B., De Gaetano G., Iacoviello L., On Behalf of the MOLI-SANI Study Investigators (2017). Mediterranean-type diet is associated with higher psychological resilience in a general adult population: Findings from the Moli-sani study. Eur. J. Clin. Nutr..

[78] Veronese N., Stubbs B., Noale M., Solmi M., Luchini C., Maggi S. (2016). Adherence to the Mediterranean diet is associated with better quality of life: Data from the Osteoarthritis Initiative. Am. J. Clin. Nutr..

[79] Crichton G.E., Bryan J., Hodgson J.M., Murphy K.J. (2013). Mediterranean diet adherence and self-reported psychological functioning in an Australian sample. Appetite.

[80] Perez-Tasigchana R.F., León-Muñoz L.M., López-García E., Banegas J.R., Rodríguez-Artalejo F., Guallar-Castillón P. (2016). Mediterranean diet and health-related quality of life in two cohorts of community-dwelling older adults. PLoS ONE.

[81] Muros J.J., Cofre-Bolados C., Arriscado D., Zurita F., Knox E. (2017). Mediterranean diet adherence is associated with lifestyle, physical fitness, and mental wellness among 10-y-olds in Chile. Nutrition.

[82] López-Gil J.F., Oriol-Granado X., Izquierdo M., Ramírez-Vélez R., Fernández-Vergara O., Olloquequi J., García-Hermoso A. (2020). Healthy Lifestyle Behaviors and Their Association with Self-Regulation in Chilean Children. Int. J. Environ. Res. Public Health.

[83] Fava G.A., Ruini C. (2003). Development and characteristics of a well-being enhancing psychotherapeutic strategy: Well-being therapy. J. Behav. Ther. Exp. Psychiatry.

[84] Ruini C., Fava G.A. (2009). Well-being therapy for generalized anxiety disorder. J. Clin. Psychol..

[85] Ruini C., Fava G.A. (2012). Role of Well-Being Therapy in Achieving a Balanced and Individualized Path to Optimal Functioning. Clin. Psychol. Psychother..

[86] Ruini C., Ryff C.D. (2016). Using eudaimonic well-being to improve lives. The Wiley Handbook of Positive Clinical Psychology.

[87] Friedman E.M., Ruini C., Foy C.R., Jaros L., Love G., Ryff C.D. (2019). Lighten UP! A Community-Based Group Intervention to Promote Eudaimonic Well-Being in Older Adults: A Multi-Site Replication with 6 Month Follow-Up. Clin. Gerontol..

[88] Sin N. (2009). Enhancing well-being and alleviating depressive symptoms with positive psychology interventions: A practice-friendly meta-analysis. J. Clin. Psychol..

[89] Bolier L., Haverman M., Westerhof G.J., Riper H., Smit F., Bohlmeijer E. (2013). Positive psychology interventions: A meta-analysis of randomized controlled studies. BMC Public Health.

[90] Weiss L.A., Westerhof G.J., Bohlmeijer E.T. (2016). Can We Increase Psychological Well-Being? The Effects of Interventions on Psychological Well-Being: A Meta-Analysis of Randomized Controlled Trials. PLoS ONE.

[91] Unützer J., Katon W.J., Fan M.-Y., Schoenbaum M.C., Lin E.H.B., Della Penna R.D., Powers D. (2008). Long-term cost effects of collaborative care for late-life depression. Am. J. Manag. Care.

[92] Mancuso C.A., Choi T.N., Westermann H., Wenderoth S., Hollenberg J.P., Wells M.T., Isen A., Jobe J.B., Allegrante J.P., Charlson M.E. (2012). Increasing Physical Activity in Patients With Asthma Through Positive Affect and Self-affirmation. Arch. Intern. Med..

[93] Ogedegbe G.O. (2012). A randomized controlled trial of positive affect intervention and medication adher-ence in hypertensive African Americans. Arch. Intern. Med..

[94] Peterson J.C., Charlson M.E., Hoffman Z., Wells M.T., Wong S.-C., Hollenberg J.P., Jobe J.B., Boschert K.A., Isen A.M., Allegrante J.P. (2012). A Randomized Controlled Trial of Positive-Affect Induction to Promote Physical Activity After Percutaneous Coronary Intervention. Arch. Intern. Med..

[95] Celano C.M. (2018). Optimizing a Positive Psychology Intervention to Promote Health Behaviors After an Acute Coronary Syndrome: The Positive Emotions After Acute Coronary Events III (PEACE-III) Random-ized Factorial Trial. Psychosom. Med..

[96] Massey C.N., Feig E.H., Duque L., Wexler D., Moskowitz J.T., Huffman J.C. (2019). Well-being interventions for individuals with diabetes: A systematic review. Diabetes Res. Clin. Pract..

[97] WHO (2019). Sustainable Healthy Diets—Guiding Principles.

[98] Mbow C. (2019). Food Security. Special Report on Climate Change, Desertification, Land Degradation, Sustainable Land Management, Food Security, and Greenhouse Gas Fluxes in Terrestrial Ecosystems.

[99] Fardet A., Rock E. (2020). Ultra-Processed Foods and Food System Sustainability: What are the Links?. Sustainability.

[100] Nelson M., Hamm M.W., Hu F.B., Abrams S., Griffin T.S. (2016). Alignment of Healthy Dietary Patterns and Environmental Sustainability: A Systematic Review. Adv. Nutr..

[101] Grosso G., Fresán U., Bes-Rastrollo M., Marventano S., Galvano F. (2020). Environmental Impact of Dietary Choices: Role of the Mediterranean and Other Dietary Patterns in an Italian Cohort. Int. J. Environ. Res. Public Health.

[102] Rosi A., Biasini B., Donati M., Ricci C., Scazzina F. (2020). Adherence to the Mediterranean Diet and Environmental Impact of the Diet on Primary School Children Living in Parma (Italy). Int. J. Environ. Res. Public Health.

[103] Vanham D., Mekonnen M.M., Hoekstra A.Y. (2020). Treenuts and groundnuts in the EAT-Lancet reference diet: Concerns regarding sustainable water use. Glob. Food Secur..

[104] González-García S., Esteve-Llorens X., Moreira M.T., Feijoo G. (2018). Carbon footprint and nutritional quality of different human dietary choices. Sci. Total Environ..

[105] Ruini L.F., Ciati R., Pratesi C.A., Marino M., Principato L., Vannuzzi E. (2015). Working toward Healthy and Sustainable Diets: The Double Pyramid Model Developed by the Barilla Center for Food and Nutrition to Raise Awareness about the Environmental and Nutritional Impact of Foods. Front. Nutr..

[106] Serra-Majem L. (2018). The Mediterranean diet as an example of food and nutrition sustainability: A multi-disciplinary approach. Nutr. Hosp..

[107] Berry E.M. (2019). Sustainable Food Systems and the Mediterranean Diet. Nutrients.

[108] FAO, FIDA, OMS, PMA, UNICEF (2020). Executive Summary of El Estado de la Seguridad Alimentaria y la Nutrición en el Mundo 2020. Transformación de los Sistemas Alimentarios para que Promuevan Dietas Asequibles y Saludables.

[109] Toti E., Di Mattia C., Serafini M. (2019). Metabolic Food Waste and Ecological Impact of Obesity in FAO World’s Region. Front. Nutr..

[110] Edwards P., Roberts I. (2009). Population adiposity and climate change. Int. J. Epidemiol..

[111] Rosi A., Mena P., Pellegrini N., Turroni S., Neviani E., Ferrocino I., Di Cagno R., Ruini L., Ciati R., Angelino D. (2017). Environmental impact of omnivorous, ovo-lacto-vegetarian, and vegan diet. Sci. Rep..

[112] Dernini S., Berry E., Serra-Majem L., La Vecchia C., Capone R., Medina F., Aranceta-Bartrina J., Belahsen R., Burlingame B., Calabrese G. (2017). Med Diet 4.0: The Mediterranean diet with four sustainable benefits. Public Health Nutr..

[113] Gastronomía y Marca País (2016). Informe Final. Gerencia de Estudios Imagen Chile. https://slideplayer.es/slide/10231701/.

[114] D’Innocenzo S., Biagi C., Lanari M. (2019). Obesity and the Mediterranean Diet: A Review of Evidence of the Role and Sustainability of the Mediterranean Diet. Nutrients.

[115] Drewnowski A., Monsivais P. (2020). Taste, cost, convenience, and food choices. Present Knowl. Nutr..

[116] Garnett T., Mathewson S., Angelides P., Borthwick F. (2015). Policies and actions to shift eating patterns: What works?. Foresight.

[117] FAO, IFAD, UNICEF, WFP, WHO (2019). The State of Food Security and Nutrition in the World 2019 Safeguarding Against Economic Slowdowns and Downturns.

[118] Saulle R., Semyonov L., la Torre G. (2013). Cost and Cost-Effectiveness of the Mediterranean Diet: Results of a Systematic Review. Nutrients.

[119] Drewnowski A., Eichelsdoerfer P. (2009). The Mediterranean diet: Does it have to cost more?. Public Health Nutr..

[120] Vlismas K., Panagiotakos D.B., Pitsavos C., Chrysohoou C., Skoumas Y., Sitara M., Yfantopoulos J.N., Stavrinos V., Stefanadis C. (2010). Quality, but not cost, of diet is associated with 5-year incidence of CVD: The ATTICA study. Public Health Nutr..

[121] Lopez C.N. (2009). Costs of Mediterranean and western dietary patterns in a Spanish cohort and their relationship with prospective weight change. J. Epidemiol. Community Health.

[122] Schröder H., Marrugat J., Covas M. (2006). High monetary costs of dietary patterns associated with lower body mass index: A population-based study. Int. J. Obes..

[123] Goulet J., Lamarche B., Lemieux S. (2008). A Nutritional Intervention Promoting a Mediterranean Food Pattern Does Not Affect Total Daily Dietary Cost in North American Women in Free-Living Conditions. J. Nutr..

[124] OECD (2019). The Heavy Burden of Obesity and the Economics of Prevention.

[125] Dalziel K.M., Segal L., De Lorgeril M. (2006). A Mediterranean Diet Is Cost-Effective in Patients with Previous Myocardial Infarction. J. Nutr..

[126] Dalziel K.M., Segal L. (2007). Time to give nutrition interventions a higher profile: Cost-effectiveness of 10 nutrition interventions. Health Promot. Int..

[127] Panagiotakos D. (2007). Estimating the 10-year risk of cardiovascular disease and its economic consequences, by the level of adherence to the Mediterranean diet: The ATTICA study. J. Med. Food.

[128] (2016). Catastro Nacional de Ferias Libres. https://www.catastroferiaslibres.cl/doc/catastro_ferias.pdf.

[129] Vilarnau C., Stracker D.M., Funtikov A., da Silva R., Estruch R., Bach-Faig A. (2018). Worldwide adherence to Mediterranean Diet between 1960 and 2011. Eur. J. Clin. Nutr..

[130] Belahsen R., Rguibi M. (2006). Population health and Mediterranean diet in southern Mediterranean countries. Public Health Nutr..

[131] Willett W., Rockström J., Loken B., Springmann M., Lang T., Vermeulen S., Garnett T., Tilman D., Declerck F., Wood A. (2019). Food in the Anthropocene: The EAT–Lancet Commission on healthy diets from sustainable food systems. Lancet.

